# Enhancing training in spiritual and religious competencies in mental health graduate education: Evaluation of an integrated curricular approach

**DOI:** 10.1371/journal.pone.0306114

**Published:** 2024-09-23

**Authors:** Michelle J. Pearce, Kenneth I. Pargament, Serena Wong, Hannah Hinkel, Sarah Salcone, Grant Morgan, Derek Kemp, Brady Brock, Esther Kim, Holly K. Oxhandler, Cassandra Vieten, Jesse Fox, Edward C. Polson, Joseph M. Currier

**Affiliations:** 1 Graduate School, University of Maryland, Baltimore, Maryland, United States of America; 2 Department of Psychiatry and Behavioral Sciences, Duke University Medical Center, Durham, North Carolina, United States of America; 3 Department of Psychology, Bowling Green State University, Bowling Green, Ohio, United States of America; 4 Department of Psychiatry, University of Western Ontario, London, Ontario, Canada; 5 Psychology Department, University of South Alabama, Mobile, Alabama, United States of America; 6 Diana R. Garland School of Social Work, Baylor University, Waco, Texas, United States of America; 7 Department of Family Medicine, University of California, San Diego, California, United States of America; 8 Department of Counselor Education, Stetson University, DeLand, Florida, United States of America; National University of Sciences and Technology, PAKISTAN

## Abstract

Despite practice guidelines for multiculturally competent care, including spiritual/religious diversity, most mental health graduate training programs do not formally address spiritual/religious competencies. Thus, we enhanced the Spiritual Competency Training in Mental Health (SCT-MH) course curriculum to train graduate students in foundational attitudes, knowledge, and skills for addressing clients’ spirituality and/or religion (S/R). The hybrid (online and in-person) SCT-MH course curriculum was integrated into existing required graduate clinical courses (replacing 15% of a course’s curriculum) and taught to 309 students by 20 instructors in 20 different graduate training programs across counseling, psychology, and social work disciplines. Using a multiple baseline waitlist control design in which students served as their own controls, students completed validated assessments at three timepoints evaluating their spiritual/religious competencies for understanding the intersection between S/R and mental health. We also collected qualitative data from the students to evaluate acceptability of the content and format of the training program. Students’ scores on all seven measures of spiritual/religious competencies had a statistically significant positive increase after engaging with the SCT-MH curriculum compared to the control period. At the end of the course, 97% of the students envisioned using spiritually integrated therapy techniques with their clients at least some of the time, 92% or more rated the materials as helpful and relevant, and 96% were satisfied with the training modules. Results demonstrate that dedicating a small (i.e., 6 hours of class time; 10 hours outside class time) but intentional amount of course time to teaching spiritual/religious competencies increases students’ attitudes, knowledge, and skills for attending to clients’ S/R in clinical practice. The SCT-MH hybrid course content is freely available to all graduate programs on our website. https://www.spiritualandreligiouscompetenciesproject.com/resources/sct-mh.

## Introduction

Despite practice guidelines and ethical mandates for multiculturally competent care, including diversity in spirituality and religion, e.g., [1–4], most mental health graduate training programs do not formally address spiritual/religious (S/R) (Note that we use the acronym S/R to indicate that spirituality and religion are different, yet interrelated concepts.) competencies in their curricula. As such, mental health professionals are not generally taught to understand nor address the deeper, spiritually-related dimensions that underlie many clients’ psychological suffering and recovery [5–8]. Further, clinicians are generally not taught how to recognize and use S/R resources that have been shown to bolster mental health [9] or to identify and address spiritual struggles that contribute to psychological distress and further hinder recovery [10–12]. However, research demonstrates that many clients view their spirituality as relevant to their mental health and also express a desire to discuss S/R issues in treatment [13]. As such, the artificial segregation of S/R experience from the training of mental health professionals ignores fundamental aspects of who clients are and how they cope, jeopardizing the quality of care we can provide them.

Fortunately, greater attention is being given to S/R dimensions of clients’ lives through the standardization and adoption of a set of S/R competencies, as well as the development of training programs based on these competencies (e.g., [11, 14, 15]). Indeed, training is the second most potent predictor of a clinician’s responsiveness to a client’s spirituality and/or religious faith in the treatment process, after their intrinsic religiousness (i.e., viewing religion as an end to itself and a master motive) [16]. Thus, training in S/R competencies for mental health care is a critical factor in improving the way mental health care is delivered and ensuring that clients’ S/R needs and concerns are addressed in treatment.

S/R competence is a form of multicultural competency [17], which includes a dynamic tripartite set of attitudes, knowledge, and skills about various religious and spiritual traditions that are developed over time [18]. Through a comprehensive literature review, working groups, and surveys of experts and clinicians, Vieten and colleagues [19, 20] identified 16 basic S/R competencies that include three attitudes, seven knowledge items, and six skills. A spiritually competent practitioner, for example, will possess awareness about how a S/R background and worldview might influence or bias their internal reactions to their clients (attitude); have an understanding about the distinct but interrelated relationships between spirituality and religion and how these areas affect mental health (knowledge); and have the ability to assess and address ways that spirituality and religion might support or cause harm to resilience, recovery, and well-being (skills).

### Current training in S/R competencies in mental health graduate programs

In recent years, researchers and mental health practitioners have articulated a compelling empirically-based rationale for the integration of S/R aspects of clients’ lives into their mental health care [14, 15, 21, 22]. These reasons include studies showing spirituality and/or religious faith can be a positive resource for many people. For example, research has shown that, in times of stress, people often look to their spirituality/religion as a way to understand and deal with their situations [23]. Similarly, according to a review of over 1,000 studies, indicators of religious beliefs, practices, and commitment have been associated with lower levels of anxiety, depression, drug and alcohol abuse, and greater well-being [9]. Of note, rates of religious coping are particularly high among older adults, people with mental health conditions, and members of minority religious and cultural groups, such as African Americans. Although spirituality and religion can be a positive resource for many, studies also show that some expressions are problematic. For example, spiritual struggles–tensions, questions, and conflicts around sacred matters with the supernatural, within oneself, and with other people–have been associated with increased risk for a variety of mental health problems, including suicidality, emotional and behavioral dysregulation, and anxiety and depression symptoms [12]. Further, other findings indicate that many clients want spiritually-sensitive mental health care [13] and that integration of a client’s spirituality and/or religious faith into care in culturally congruent ways can support the effectiveness of treatment [21]. Adequate training and education in S/R competence and mental health care is necessary to address each of the above issues and cultivate clinical competence to be responsive to many clients’ spiritual and/or religious identities.

Yet, despite the clinical and cultural relevance of spirituality and/or religious faith, most mental health disciplines offer limited training along these lines. According to the most recent data available, only 25% of clinical psychology training programs [8] and 30% of social work programs [24] provide a course on spirituality/religion and mental health, most of which are not required and some of which are housed in other departments, such as anthropology. Further, a survey study by Schafer and colleagues [8] revealed that among the clinical psychology programs that do offer a course on S/R competence, these courses are offered variably and only a quarter of them are required; for the most part, courses on S/R competence in psychology programs are electives. The topic of spirituality/religion is most often addressed in supervision (84%) or in an unrelated course (76%), such as multicultural diversity or ethics and professional issues. Of note, only 39% of the Directors of Clinical Training in Psychology in Schafer et al.’s [8] sample responded to the survey and schools with a religious affiliation were more likely to report systematically covering S/R competence in their programs. Thus, the representation of spirituality/religion in psychology programs may be overstated.

In addition, an evaluation of S/R diversity training in APA-accredited psychology doctoral programs and pre-doctoral internships, assessing 292 students, interns, faculty, and training directors (55% response rate) revealed that among the various areas of diversity, spirituality/religion were given the least attention, and such efforts were rated the least effective in developing competence [25]. This study also found that psychology programs have been relying on informal and unsystematic sources of learning to provide training. Notably, the most frequent sources of learning identified were clinical experiences and interaction with peers. This suggests a reactive rather than proactive stance toward training in S/R competence, one that overlooks the considerable body of research and practical literature on the interface between S/R and mental health. A similar dearth of targeted S/R education/training has also been reported among professional counselors and marriage and family therapists [7, 26]. One notable exception to this rule comes from psychiatry, which often requires training in S/R competence as part of U.S. residency programs [27].

Given the paucity of training, it is not surprising that very few studies have evaluated the effectiveness of mental health training programs in fostering competencies in spirituality/religion as core areas of diversity and psychological functioning. For instance, one six-hour training initiative for psychiatry residents reported increases over the course of the program in S/R competencies and comfort in dealing with spirituality [27, 28]. Additionally, our research group developed and evaluated an eight-hour online S/R competence training program (SCT-MH) for 169 mental health professionals from diverse disciplines [14, 29]. Participants demonstrated significant increases in S/R competencies involving attitudes, knowledge, and skills based on Vieten et al.’s [19] work in this area. For example, providers reported an increase in attending to how their own S/R background may influence their clinical practice (attitude), knowing spiritual beliefs and practices that support or hinder well-being (knowledge), and inquiring about a client’s S/R in their assessment (skill). Participants also reported high levels of satisfaction with the program and greater likelihood of integrating their clients’ spirituality/religion into treatment when indicated. These findings were recently replicated with another sample of 173 mental health providers [30]. These studies, albeit preliminary, suggest that it is possible to foster S/R competence in mental health care through formal and intentional training efforts.

### Description of the SCT-MH integrated graduate curriculum

There is a pressing need to bridge the gap between effective methods of training in foundational facets of S/R competence and the current deficit in graduate education across mental health disciplines in these core areas of multicultural diversity. By starting at the outset of graduate coursework, we can begin to equip a new generation of educators, clinicians, and supervisors with S/R competence. To address this training need, we modified the program we developed for mental health providers for graduate students [14, 29, 30]. Namely, the goal of the integrated Spiritual Competency Training in Mental Health (SCT-MH) hybrid (online and in-person) graduate course curriculum is to equip students across mental health disciplines with greater confidence and competence in attending to clients’ possible spiritual/religious identities in their clinical work. Notably, the program did not have a theological orientation and instead was intentionally designed to train therapists to work with clients across all religious/spiritual and non-religious/spiritual beliefs, practices, values, and identities and, thus, is applicable to all clients and R/S identities. The content is designed to be easily integrated into Master’s and doctoral level clinical graduate courses, representing about 15% of the content in a typical semester-long clinical course. The online and in-person curriculum was developed by the online course directors (MP and KP) by drawing upon educational materials on spiritually integrated psychotherapy and S/R competencies that have been disseminated over the last 15 years (e.g., [11, 15, 31, 32]. All course materials and activities were designed to promote the three dimensions of S/R competence (attitudes, knowledge, skills) and were mapped to Vieten et al.’s [19] 16 basic competencies. Notably, this integrated curriculum approach did not require instructors to become experts in spirituality/religion or add another stand-alone course to a program’s usually full curriculum. This approach also closely mirrors how S/R is taught in medical and psychiatry programs: only 7% of medical schools have a required course on S/R, whereas 73% of medical schools include S/R content in required courses addressing other topics [33].

### Study aims

Our study goal was therefore to evaluate whether an enhanced hybrid version of the existing SCT-MH training program can be widely integrated by graduate school instructors into existing clinically-related courses in a manner that results in significant changes in students’ S/R competencies, both self-reported and based on objective content knowledge. Drawing on a multiple-baseline design in which students completed the standardized integrated curriculum during a six-week period after being tracked for a six-week period without any intervention, we proposed two primary hypotheses:

H1: When compared to changes in the six-week period without any intervention, students will report significantly greater improvements in S/R competence scores after completing the SCT-MH content; specifically, we hypothesize that scores on self-report and objective measures of attitudes, knowledge, and skills will increase from pre- to post-training.
H2: The integrated curriculum approach will be a feasible, helpful, and relevant way to deliver training on S/R competencies in mental health care via required coursework in graduate programs, as demonstrated by responses of students across quantitative and qualitative assessments in the study.

## Methods

### Transparency and openness

We report how the sample size was determined, all data exclusions, manipulations, and measures in the proceeding sections. This study’s design, hypotheses, and analytic plan were registered on the Open Science Framework (OSF) before collecting the data; analysis code, questionnaires, and other materials are available at: https://osf.io/7qdgn/?view_only=f2768396243d4b93b4bdf5167e6ecc68.

### Sample description

This study focused on faculty instructors and students from 20 graduate training programs in counseling, psychology, and social work (for details about these programs, see Table 1). The instructors were selected from a request for proposals (RFP) competition issued in January 2022 for graduate faculty from these professions to design a plan for integrating an enhanced hybrid version of the SCT-MH program into a required course in their training program that would be taught in the spring term of 2023. Drawing upon a larger grant from the John Templeton Foundation, selected instructors were awarded funding in August 2022 to support their involvement over the 2022–2023 academic year, including completing the SCT-MH program themselves, replacing 15% of their existing course content with the standardized hybrid content, teaching the content to their graduate students according to the study stipulations, and assisting with the research procedures described below. Instructors also participated in a one-day virtual training session before the course began and three consultation calls during the implementation period focused on developing proficiency with teaching the enhanced version of the SCT-MH program in their courses and settings.

**Table 1:** Overview of training programs and SCT-MH implementation contexts.

Institution	Profession	Degree	Course (Required in Curriculum)	Number of Students (N = 268)
Albizu University	Psychology	Psy.D.	Diversity and Culture in Clinical Practice	24
Alfred University	Counseling	MS Ed.	Topics in Counseling	6
Chatham University	Psychology	Psy.D.	Practicum III	8
Indiana University	Social Work	MSW	Mental Health and Addictions Practice: Individuals and Families	12
Long Island University	Counseling	M.S.	Clinical Mental Health Counseling Internship II	10
Louisiana State University in Shreveport	Counseling	M.S.	Counseling Internship II	11
Loyola University Maryland	Psychology	Psy.D.	Advanced Psychopathology Seminar	17
New Mexico Highlands University	Counseling	M.A.	Internship in Clinical Mental Health Counseling	10
Rutgers University	Psychology	Ph.D.	Motivation and Emotion: Biological and Affective Basis of Behavior	19
SUNY Brockport	Social Work	MSW	Cultural Humility and Social Work Practice	15
University of California, Berkeley	Psychology	Ph.D.	Professional Development in Clinical Science	8
University of Central Florida	Counseling	M.A.	Human Sexuality & Relationships	19
University of Hawai’i at Mānoa	Social Work	MSW	Advanced Practice In Behavioral Mental Health	17
University of Missouri–St. Louis	Social Work	MSW	Advanced Social Work Practice Across The Lifespan	7
University of North Florida	Counseling	M.S.	Introduction to Family Counseling	24
University of South Alabama	Counseling	M.S.	Addictions Counseling	9
University of Southern Mississippi	Psychology	M.S.	Field Internship	12
University of Texas Rio Grande Valley	Counseling	M.Ed.	Clinical Internship II	7
University of West Florida	Social Work	MSW	Advanced Year Field Instruction and Integrative Seminar I	8
Utah State University	Social Work	MSW	Advanced Generalist Social Work Practice II: Couples, Families, & Group	25

The average age of the faculty instructors was 45.40 years (*SD* = 6.76) and the gender distribution was 50% cisgender female, 45% cisgender male, and 5% non-binary. In total, 75% of this group identified as White or Caucasian and 25% identified as Black or African American, Hispanic or Latinx, or Asian or Pacific Islander. Religious affiliations ranged from Christian (Catholic = 30%, Protestant = 30%) to other religions (e.g., Jewish; 25%) and non-affiliated (15%). Half of the instructors identified as “moderately” or “very” religious and all but one viewed themselves as a “moderately” or “very” spiritual person. All of the instructors held a doctorate degree in their field and 80% were licensed to practice clinically. They held these positions in their training programs at the time of the study: Director of Clinical Training = 10%, Full Professor = 15%, Associate Professor = 35%, Assistant Professor = 35%, Associate Clinical Professor = 5%. Half of the instructors had been teaching for 10 or more years and 60% had not received any formal training in S/R competence before the study.

The recruitment of students for this study began on January 1, 2023 and ended on May 30, 2023. In total, 351 students registered for the instructors’ selected courses in the Spring 2023 term in which the enhanced SCT-MH program would be included (for details about the courses and relative numbers of students, see Table 1). These students were invited via email to participate in evaluation surveys administered via Qualtrics consisting of validated measures of S/R competence and other assessments at Week 1 (Baseline), Week 6 (Start of SCT-MH Program), and Week 12 (End of SCT-MH Program) of the course. Although students needed to complete the SCT-MH modules, activities, and assignments for their course requirements, they could abstain from completing the surveys without affecting their grade or status in the course. Further, instructors were not notified about whether their students voluntarily opted to participate in the research procedures and were not provided access to the resulting dataset. Students who opted to complete the research surveys first signed a written consent form, which was part of the Qualtrics survey packet, before completing the first survey. Of the overall group of students, 309 completed the baseline assessment (88% response rate). Of this sample, 268 students completed the study assessments again at Time 2 and 3 (86.7% retention rate).

The average age in the final sample of students was 30.16 years (*SD* = 8.22) and gender distribution was 84.7% cisgender female, 13.8% cisgender male, and 1.4% transgender or non-binary. Students reported these rates of racial/ethnic identities: White or Caucasian = 61.2%, Hispanic or Latinx = 16.4%, Black or African American = 9.7%, Asian or Pacific Islander = 6.0%, and other groups = 6.6%). Nearly half of the sample identified as Christian (Catholic = 13.1%, Orthodox = 0.4, Protestant = 33.6%); roughly one-fifth were affiliated with Judaism (5.6%), Buddhism (2.6%), and other organized religions (12.2%); and one-third of students identified as non-affiliated (33.6%). Less than one-third (30.6%) of the students identified as “moderately” or “very” religious and 61.9% viewed themselves as a “moderately” or “very” spiritual person. All of the students were in their second-year or beyond in the training program and were engaged in a clinical practicum or internship that involved supervised practice with clients. In total, 54.9% of the students had not received prior training in integrating their clients’ S/R into clinical practice in coursework, workshops, or field/clinical work before the study.

### Overview SCT-MH course

The Spiritual Competency Training in Mental Health (SCT-MH) hybrid graduate course curriculum consists of two parts: (1) a minimally modified version of the existing eight online modules housed on the edX platform (i.e., ~10 hours of asynchronous, self-paced multimedia content and learning activities) (Note: The original online SCT-MH program for mental health providers is described in Pearce et al. [14]); and (2) five 30-minute class discussions and one class devoted to role-playing skill development for S/R assessment (~3 hours). We designed the curriculum for the in-person weekly classes using the flipped classroom approach. Specifically, students engage with all learning resources (e.g., readings, videos, case studies) before attending class. Class time is then devoted to discussions and skill building activities, such as role plays. The flipped classroom approach addresses the current limitation in most multicultural diversity courses, which prioritize the formation of attitude and knowledge-based competencies over acquisition of skills [Priester PE et al. [Unpublished]]. Key topics included: common stereotypes about spirituality/religion; the diversity of S/R forms and expressions; important reasons for addressing spirituality/religion in treatment; the importance of the therapist’s own S/R attitudes, beliefs, and practices; how to assess clients’ spirituality/religion; how to help clients access S/R resources; and how to respond to S/R problems that arise in treatment. Modifications to Pearce et al.’s [14] original SCT-MH program for practicing clinicians included the addition of video case critiques, more case examples, suggested clinical practice activities with follow-up reflection, and a graded final case study based on Vieten et al.’s [19, 20] competencies (see Table 2 for a description of the SCT-MH training program modules).

**Table 2:** Description of SCT-MH training program modules.

Training Program Module	Description	Spiritual/Religious Competencies Addressed in Module
*Module 1*: Introduction and Orientation	What is spiritually integrated mental health care? Why integrate spirituality into therapy? What does it take to do spiritually integrated therapy?	#3. Being aware of your own beliefs: Attitude #10. Being aware of legal and ethical issues: Knowledge #15. Staying up-to-date: Skill
*Module 2*: Understanding Spirituality	Defining spirituality and religion: Similarities and differences Religious and spiritual diversity Spiritual development across the lifespan and the forces that influence this process	#5. Understanding spirituality and religion as different but overlapping: Knowledge #7. Recognizing spiritual and religious development over lifespan: Knowledge #8. Learn about diverse beliefs and practices: Knowledge
Module 3: Guiding Principles for Spiritually Integrated Mental Health Care	Inappropriate therapist orientations to spirituality in mental health Effective therapist orientation to spiritually competent care The therapists’ own spiritual orientation and spiritual biases	#1. Demonstrating empathy, respect, and appreciation: Attitude #2. Appreciating spiritual and religious diversity: Attitude #3. Being aware of your own beliefs: Attitude
*Module 4*: Distinguishing between Helpful and Harmful Types of Spirituality	Life-affirming, helpful forms of spirituality Life-limiting, unhelpful forms of spirituality Distinguishing between spiritual experiences and psychopathology?	#6. Difference between spirituality and psychopathology: Knowledge #8. Learn about clients’ spiritual and religious resources: Knowledge #9. Recognize harmful spiritual and religious involvement: Knowledge
*Module 5*: Assessing Spirituality in Mental Health Care	Setting the stage for spiritual assessment Initial, implicit, and explicit spiritual assessment	#11. Working with spiritual and religious diversity: Skill #12. Conducting spiritual and religious assessment: Skill
*Module 6*: Assessing and Mobilizing Spiritual Resources	Guidelines for integrating spiritual resources into therapy Cultivating and mobilizing spiritual resources	#8. Learn about clients’ spiritual and religious resources: Knowledge #13. Helping clients identify/ access spiritual and religious resources: Skill
*Module 7*: Assessing and Addressing Spiritual Problems	What not to do when encountering spiritual problems How to address spiritual problems in therapy Addressing spiritual problems	#9. Recognize harmful spiritual and religious involvement: Knowledge #14. Helping clients identify and deal with spiritual and religious problems: Skill #16. Acknowledging limits: Skill
*Module 8*: Putting it All Together, Challenges, and Future Directions	Ethical concerns of spiritually integrated therapy Synthesize and apply knowledge from all eight modules to a hypothetical clinical case planning decision making Summing up the program	#10. Develop awareness of legal and ethical issues: Knowledge #15. Stay up-to-date: Skill #16. Acknowledging limits: Skill

To promote skill proficiency, class meetings incorporated topical discussions and role-playing exercises to gain exposure and comfortability with basic clinical practices for spiritually responsive care (e.g., S/R assessment, integration of S/R content into treatment planning and psychosocial interventions, collaborating with clergy). Consistent with the multicultural competence literature [34], students also engaged in reflective exercises to enhance self-awareness of their own attitudes/beliefs about S/R. Each of the instructors were given a standardized curriculum, syllabus, and guidebook with copies of the class activities, as well as some flexibility in implementation. Specifically, a portion of the face-to-face time each week was set aside to discuss issues and topics most relevant to their students’ training needs, as well as features of training in their discipline.

### Measures

#### S/R competence measures

Students completed the below measures at Week 1, Week 6, and Week 12 of their courses to evaluate possible changes in varying facets of S/R competence:

*Religious/Spiritually Integrated Practice Assessment Scale (RSIPAS)* [35]. Three of the four RSIPAS subscales were included in the current study to assess students’ self-reported competencies in one attitude domain (Attitudes about S/R integrated clinical practice; 12 items; range 12–60), and two skills domains (Self-Efficacy with S/R integrated clinical practice; 13 items; range 13–65; and Current Engagement in S/R integrated practice; 9 items; range 9–45). Sample items include: “It is essential to assess clients’ religious/spiritual beliefs in practice” (Attitude); “I am comfortable discussing my clients’ religious/spiritual struggles” (Self-Efficacy); and “I use empirically supported interventions that specifically outline how to integrate my clients’ religion/spirituality into treatment” (Current Engagement). Items were rated on a 5-point scale in which 1 = *Strongly disagree* and 5 = *Strongly agree*, such that higher scores indicated greater perceived S/R competence. The RSIPAS has demonstrated strong internal consistency, convergent/divergent validity, and other psychometric properties in a number of studies (e.g., [29, 30, 35]. Cronbach’s alphas fell in these ranges for the three subscales across the longitudinal assessments: Attitudes = .86-.87; Self-Efficacy = .90-.91; Engagement = .86-.88.

*Spiritual Competency Questionnaire (SCQ)* [29]. Drawing upon Vieten et al.’s [20] competencies, the 16-item SCQ assessed students’ perceived Attitudes (3 items; range = 3–21), Knowledge (6 items; range = 6–42), and Skills (7 items; range = 7–49) related to attending to clients’ S/R in their practice. Sample items include: “I make a conscious, daily effort to respect clients from diverse backgrounds (spiritual, religious, or secular)” (Attitudes); “I know how to tell the difference between spiritual experiences and psychopathological symptoms” (Knowledge); and “I inquire about clients’ religion and/or spirituality as a standard part of my assessment process” (Skills). Items were rated on a 7-point scale in which 1 = *Not at all true of me* and 7 = *Completely true of me*, such that higher scores again indicated greater perceived S/R competence. The SCQ has similarly displayed strong psychometric properties in previous studies [29, 30]. Cronbach’s alphas fell in these ranges for the three subscales across the three longitudinal assessments in this study: Attitudes = .67-.72; Knowledge = .83-.85; Skills = .83-.88.

*Religious and Spiritual Knowledge Questionnaire (RSKQ)*. A 21-item objective measure of the students’ basic knowledge of S/R content with relevance to spiritually responsive clinical practice was also implemented at each time point. In other words, unlike the items for self-assessment of perceived S/R competence, these items offered objective measures of the students’ knowledge. Drawing upon the parallel forms of the RSKQ that were developed and successfully used in Pearce et al.’s [29] initial evaluation study, we created a third set of items that mapped onto these existing versions for this study. We piloted the overall pool of items with 20 graduate students in a PhD program in clinical/counseling psychology or Master’s program in clinical mental health counseling at the [MASKED REVIEW] to ensure the three forms were equivalent in their difficulty-level. Namely, in addition to comparing the total scores across the three sets of items, we also reviewed item-total correlations and percentages of correct responses on each item. In turn, we eliminated two problematic items from each version and switched others to ensure the three versions of the RSKQ had adequate content validity and generated the same average total score among students in the pilot survey (Mean = 10). Items were presented in a multiple-choice format in which the students needed to identify the correct response from four choices. After scoring each item as 0 = *Incorrect* or 1 = *Correct*, we summed the total number of correct items to provide an overall index of the students’ objective S/R knowledge at each assessment for the analyses (range = 0–21).

### Instructor-graded final case assessment

We also included an instructor-graded assessment at the end of the online course to provide a multiple-rater measurement of students’ acquisition of the foundational attitudes, knowledge, and skills related to S/R competence. The ten-question final case study in the SCT-MH program was designed to assess the 16 competencies taught in the curriculum. To ensure standardization of grading across instructors, we developed a specific evaluation rubric for the case study along with an answer key, trained the instructors how to use both tools during a half-day training using hypothetical student responses, reviewed the use of the rubric during the consultation call prior to their grading of the final case studies, and answered instructors’ grading questions as they arose. As such, the students’ final grade on this case study provided a proxy measure of S/R competence achievement that we will also report in this paper.

### Acceptability questions

We also gathered quantitative and qualitative evaluative feedback from the students in the third assessment about the acceptability of varying components and dimensions of the enhanced SCT-MH program. In addition to addressing the second hypothesis of this study, data from these assessments was used to further improve the content, organization, and presentation of the enhanced SCT-MH program for future implementation in mental health graduate education.

### Research procedures

Students were invited to participate in three evaluation surveys over a 12-week period during their courses: Week 1 (Baseline), Week 6 (Immediately before starting the SCT-MH Program), and Week 12 (End of SCT-MH Program). Written consent was obtained from the students before completing the first survey. Using the same 12-week schedule across the training programs, each instructor refrained from presenting any material related to S/R for the first six weeks of the course. At Week 7, the instructors introduced the SCT-MH program and students were granted access to the online modules. In six classes over the next six weeks, the students completed the online modules outside of class and participated in structured discussions (2.5 hours total) and role plays (3 hours total) during the meetings with their instructors and classmates that were outlined in the instructor guidebook. At Week 12, students were then invited to complete the assessment battery for a third time and instructors provided the research team with the students’ SCT-MH final case study grades. In so doing, this multiple baseline design utilized changes on S/R competence measures between Week 1 to Week 6 as a no-treatment comparison condition that could be compared to the magnitude of anticipated improvements in S/R competence during the six-week period in which students completed the enhanced SCT-MH program (i.e., Week 7 to Week 12). In summary, all students in each of the 20 classes were required to complete the SCT-MH course curriculum, but only students who elected to and consented to participating in the research completed the surveys. Surveys were distributed via Qualtrics, and students received a gift card for each survey that was completed ($50 for Survey 1; $60 for Survey 2; $75 for Survey 3). Following review and approval of these research procedures, the University of South Alabama Institution Review Board (IRB) approved this study and served as the primary IRB for the study; secondary approvals, when desired, were granted from the instructors’ institutions before data collection began.

### Plan of analysis

Following our screening of the data and calculation of descriptive statistics and effect sizes for changes in S/R competence measures, we performed a series of analytic steps culminating with the estimation of a multi-factor latent growth model of the seven measures of S/R competence (attitudes, knowledge, and skills). To this end, we first computed the subscale scores for each measure in accordance with the previously published literature on the RSIPAS or SCQ. For the RSKI instrument, we computed the sum score of the number of correct responses provided for each student. Next, we generated descriptive statistics for each of these measures across time to examine the average change trajectories across the three assessment points.

Based on the observed growth patterns, we specified a piecewise latent growth trajectory for each S/R competence outcome such that the change from Time 1 (Baseline) to Time 2 (Pre-Training) was modeled separately from Time 2 (Pre-Training) to Time 3 (Post-Training). This approach estimated the stability of the S/R competencies prior to completing the SCT-MH training program (i.e., waitlist control period) and a more accurate effect of the training program. The variance for each facet of S/R competence was constrained to be equal across time, but variances were freely estimated across them. For each outcome, the covariance between the intercept and the control period slopes was fixed to zero but was freely estimated for the intercept and training slope. The covariances between intercepts and the covariances between the training slopes were freely estimated; that is, the model accounted for the correlation between the outcome at baseline as well as the SCT-MH training program’s probable effects. The covariances between the S/R competence outcomes at the end of the study were also freely estimated. The model was estimated in the lavaan package [36] in R [37] using maximum likelihood estimation, with statistical significance set as a p-value of less than .05. Last, the correlations were calculated between each of the outcomes with the grades from the end-of-course case study assignment.

Two sets of analyses were performed to examine the acceptability of infusing the enhanced SCT-MH program into the graduate course. First, frequency analyses were used to summarize quantitative feedback provided by students. Second, narrative responses to the qualitative items were examined via an inductive content analysis [38] to identify the primary themes. The authors DK and BB (both Christian White men in their 20s) coded the responses to the five questions in a manner that was blinded to students’ backgrounds or responses on other study measures. In turn, a consensus method was then used by the authors DK, BB, and JC to resolve discrepant ratings.

## Results

### Preliminary analyses

We initially screened our data for missing values, normality assumptions, and univariate and multivariate outliers. Prior to running our primary analyses, we also calculated descriptive statistics for observed scores on the outcomes measures along with effect sizes for changes in the S/R competence measures across the two six-week intervals between the assessments (see Table 3). With the exception of an increase in engagement on the RSIPAS (paired samples *t* [267] = 2.01, *p* < .023; Cohen’s *d* = 0.123) and perceived skills on the SCQ (paired samples *t* [267] = 2.28, *p* < .012; Cohen’s *d* = 0.139), students did not report improvement in the other outcomes from Week 1 to Week 6 (paired samples *t* [267] = -33.87 to 0.90, *p* < .413; Cohen’s *d* = -2.07 to 0.02). In contrast, when compared to students’ mean scores on the S/R competence measures at Week 6, they reported significantly higher scores at Week 12 (paired samples *t* [267] = 9.20 to 56.67, *p* < .001; Cohen’s *d* = 0.562 to 3.462).

**Table 3:** Means and standard deviations of the R/S competency outcomes (N = 268).

	Week 1	Week 6	Week 12	Week 1–6: Cohen’s *d*	Week 6–12: Cohen’s *d*
RSIPAS–Attitudes	50.04 (6.10)	49.78 (6.18)	53.47 (5.62)	-0.055	0.667***
RSIPAS–Self-Efficacy	40.59 (8.82)	27.94 (5.83)	52.66 (6.31)	-2.070***	3.462***
RSIPAS–Engagement	27.17 (6.59)	27.90 (6.60)	33.62 (5.92)	0.123*	0.946***
SCQ–Attitudes	18.13 (2.67)	18.01 (2.61)	19.35 (1.96)	-0.047	0.562***
SCQ–Knowledge	30.31 (7.82)	30.23 (7.68)	40.82 (5.03)	-0.013	1.37***
SCQ–Skills	20.49 (7.52)	21.37 (7.31)	29.49 (7.11)	0.139**	1.06***
R/S Knowledge Questionnaire	10.73 (2.45)	10.79 (2.25)	13.95 (3.33)	0.017	0.946***

*p < .05,

**p < .01,

***p < .001.

R/S, religion and spirituality; RSIPAS, Religious/Spiritually Integrated Practice Assessment Scale; SCQ, Spiritual Competence Questionnaire.

### Effectiveness of enhanced SCT-MH program

Overall, each of the attitude-, knowledge-, and skill-based aspects of S/R competence followed the same general pattern; that is, during the control period, the outcomes remained relatively constant followed by a significant increase following the SCT-MH training program. The only exception to this pattern was the self-efficacy outcome on the RSIPAS, which showed a noticeable decrease during the control period followed by an increase nearly double the other competency-based assessments over the next six weeks. The pattern of change trajectories for each facet of S/R competence provided descriptive evidence of the training program’s effect.

**Fig 1:**
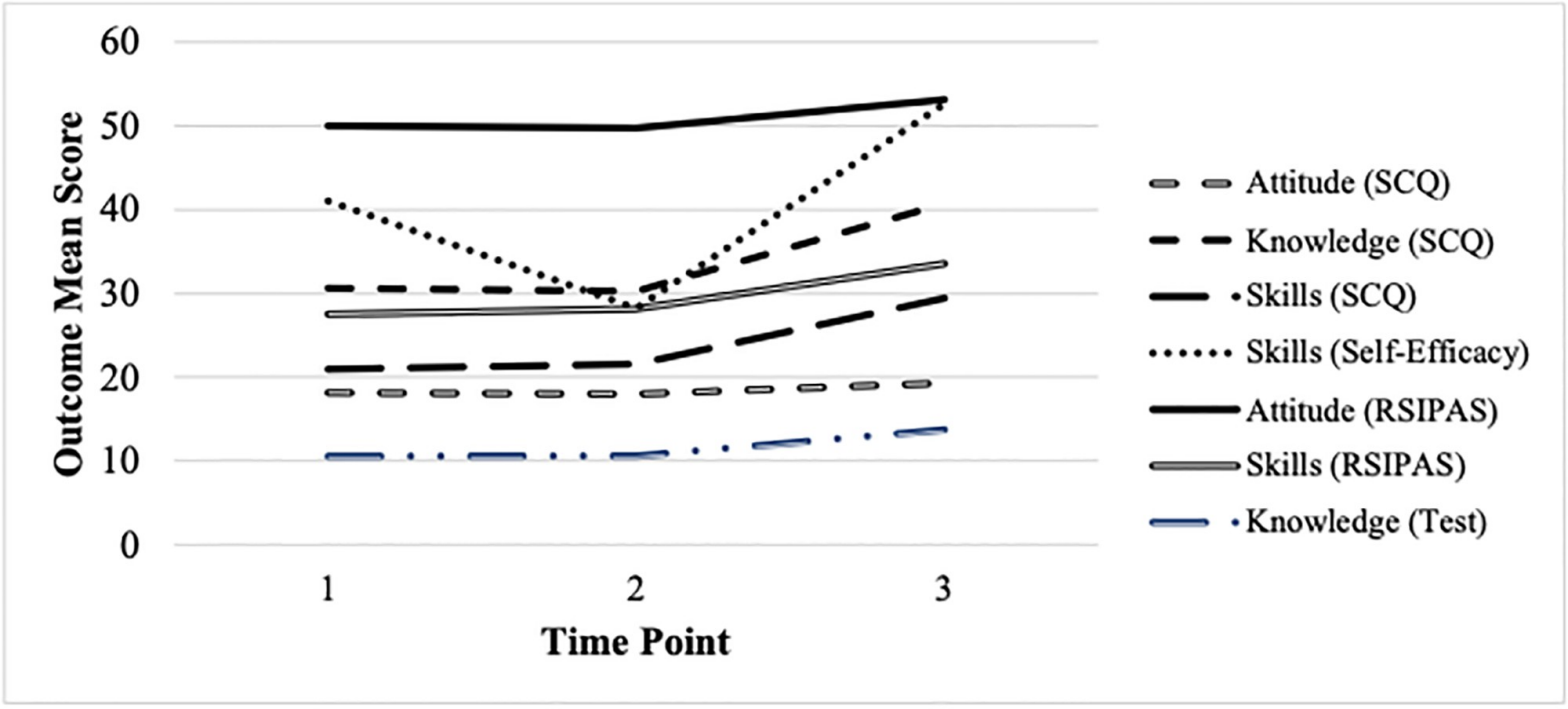
Changes in spiritual and religious competency outcomes over time. *Note*. Changes from Baseline to Start of SCT-MH Program were statistically significant for Skills (SCQ; *Z* = 2.23, *p* = .03), Skills (RSIPAS; *Z* = 1.99, *p* = .05), and Skills (Self-Efficacy; *Z* = -33.84, *p* < .001). All changes from Start of SCT-MH Program to End of SCT-MH Program were statistically significant (*p* < .001). Timepoints: 1 = Baseline; 2 = Week 6; 3 = Week 12/13.

The latent variable model of the collective growth of the seven outcomes provided additional support and insights into the SCT-MH training program’s possible effects (see Fig 1). The estimates from the growth model are shown in Table 4. The control period changes indicated by the first slopes in the model were not found to differ significantly from zero for four of the seven competencies. Two of the competencies showed increases that were statistically significant, although the observed increase was less than one point on the scaled score metric. As noted above, the self-efficacy scale on the RSIPAS showed a large decline during the control period. All increases following the training program were positive and statistically significant (*p* < .001 for all slopes). These findings indicate that all S/R competencies related to attitudes, knowledge, and skills increased after completing the training program. The standardized growth parameters on the RSIPAS competencies were .88 for Attitudes, 5.32 for Self-Efficacy, 3.06 for Engagement; competencies assessed by the SCQ were .71 for Attitudes, 2.23 for Knowledge, and 1.86 for Skills; the S/R Knowledge Questionnaire was 1.95.

**Table 4:** Latent growth model parameters estimates for spiritual competency measures.

**Fixed Effect Estimates**						
	*Intercept*		*Slope 1*		*Slope 2*	
**Competency**	Estimate	SE	Estimate	SE	Estimate	SE
RSIPAS–Attitudes	50.04*	.37	-0.26	.29	3.69*	.38
RSIPAS–Self-Efficacy	40.49*	.45	-12.65*	.37	24.72*	.37
RSIPAS–Engagement	27.18*	.40	0.73*	.36	5.71*	.36
SCQ–Attitudes	18.13*	.16	-0.12	.15	1.34*	.15
SCQ–Knowledge	30.31*	.43	-0.08	.36	10.59*	.46
SCQ–Skills	20.49*	.45	0.86*	.39	8.12*	.47
S/R Knowledge Questionnaire	10.74*	.14	0.05	.18	3.16*	.20
**Variance Estimates**						
	*Intercept*		*Slope 1*		*Slope 2*	
**Competency**	Estimate	SE	Estimate	SE	Estimate	SE
RSIPAS–Attitudes	25.09*	2.52	0.00	--	17.72*	3.11
RSIPAS–Self-Efficacy	34.81*	3.63	0.00	--	21.59*	4.63
RSIPAS–Engagement	24.64*	2.90	0.00	--	3.48	3.37
SCQ–Attitudes	3.54*	0.39	0.00	--	0.00	--
SCQ–Knowledge	32.23*	3.11	0.00	--	22.54*	4.22
SCQ–Skills	35.27*	3.87	0.00	--	19.03*	4.80
S/R Knowledge Questionnaire	1.46*	0.35	0.00	--	2.64*	0.92

* *p* < .05. S/R = Spirituality and Religion; RSIPAS = Religious/Spiritually Integrated Practice Assessment Scale; SCQ, Spiritual Competence Questionnaire.

Our model also included covariances between the intercepts and slopes in the model. The full set of output for growth parameter covariances can be found in the Supplemental Materials; however, the overall conclusion from the covariance analysis is that growth rates after the training were positive and statistically significant for all spiritual/religious competencies. In other words, the growth in one of the S/R competencies was generally associated with favorable growth in the other outcomes.

Finally, we examined the association between the change score (Time 1 to Time 3) for each of the competencies and the final case study grade at the end of the SCT-MH training program. All of the zero-order correlations, with the exception of two of the skills measures (SCQ skills and RSIPAS skills), were significantly positively correlated, although they were mostly small in magnitude (.05 to .20). The correlation matrix can be found in the Supplemental Materials.

### Acceptability of enhanced SCT-MH program

Table 5 summarizes relative frequencies of students’ responses to quantitative items in the last survey assessing acceptability of the enhanced SCT-MH program. In total, 38.5% of the sample envisioned using spiritually integrated therapy techniques at least “some of the time” with their clients and 58.5% envisioned doing so “often” or “very often”. In total, roughly 60–70% of the sample also rated the online modules, face-to-face discussions, in-class role-playing exercises, and modeling of skills as “very helpful,” or “extremely helpful” and another 22–32% rated these components as “somewhat helpful.” Similarly, nearly of the students rated the relevance of the SCT-MH training activities (online and face-to-face) and end-of-module suggestions in highly favorable ways and roughly one-third viewed them as “somewhat relevant.” Further, students rated their satisfactions with the online training modules and videos in similar ways. Nearly three-quarters of the sample also rated the amount of time needed to complete the online program and amount of material as “just right.”

**Table 5:** Frequencies of responses to quantitative acceptability questions.

	**Never**	**Rarely**	**Some of the time**	**Often**	**Very Often**
*I envision using spiritually integrated therapy techniques with my clients*.	0.3%	2.7%	38.5%	41.9%	16.6%
	**Not at all helpful**	**Not very helpful**	**Somewhat helpful**	**Very helpful**	**Extremely helpful**
*How helpful was the* *online* *SCT-MH training program in preparing you to engage in spiritually integrated therapy*?	0.3%	1.4%	26.7%	52.0%	19.6%
*How helpful were the face-to-face classroom discussions in preparing you to engage in spiritually integrated therapy*?	1.0%	4.1%	31.1%	45.3%	18.6%
*How helpful were the in-class role playing exercises in preparing you to engage in spiritually integrated therapy*?	1.7%	5.7%	31.8%	43.2%	17.6%
*How helpful was the modeling of spiritual competency skills in the online program (e*.*g*., *case studies*, *video critiques)*?	0.7%	3.0%	22.3%	54.1%	19.9%
	**Not at all relevant**	**Not very relevant**	**Somewhat relevant**	**Very relevant**	**Extremely relevant**
*How relevant was the SCT-MH training (online and face-to-face activities) to your clinical work*?	0.7%	7.4%	33.4%	44.3%	14.2%
*How relevant were the end-of-module suggestions for clinical skill application (practicing the skills with your clients) in the online training program*?	0.7%	5.1%	36.5%	42.6%	15.2%
	**Not at all satisfied**	**Not very satisfied**	**Somewhat satisfied**	**Very satisfied**	**Extremely satisfied**
*How satisfied were you with the online training program*?	0.7%	3.7%	34.5%	45.9%	15.2%
*How satisfied were you with the videos in the online training program*?	0.7%	4.1%	36.8%	44.9%	13.5%
	**Far too little**	**Moderately too little**	**Just right**	**Moderately too much**	**Far too much**
*How would you rate the amount of time needed to complete the online program*?	0.3%	3.7%	70.9%	24.0%	1.0%
*How would you rate the amount of material in the online training*?	0.3%	6.8%	72.3%	20.3%	0.3%

Table 6 presents the qualitative questions and the primary themes that emerged from the inductive content analysis. When asked about strengths of the enhanced SCT-MH program, nearly half of the students discussed the online materials (e.g., videos, reflection questions, case study); for example, one participated stated: “*I liked the videos*, *I found it very helpful to listen to real time responses to clients dealing with spiritual issues*. *I am a visual learner so that was great*. *I also appreciate the print outs*. *I found them to be very helpful and will take them to work and use them for reference*.” Similarly, 20–26% of students thought the role plays were helpful, enjoyed the class discussions to learn from their peers and faculty instructors, and/or discussed appreciation for framing S/R competence as a facet of multicultural competence and increased knowledge about S/R integration in mental health care. For example, one student stated: “*It’s breaking through the taboo*. *We’re conditioned to avoid this topic*, *but it is an integral part of the human experience that our clients do and will continue to rely upon in varying ways*. *If we are to be truly effective in improving people’s quality of life*, *incorporating spirituality*, *if that is something that they desire*, *is vital to that*. *I am grateful to have been a part of this*.”

**Table 6:** Frequencies of qualitative themes.

	Frequency
*What did you like about this spiritual competency training (online and face-to-face class activities)*?	
Materials covered throughout the training program	123 (45.9%)
Role plays to practice S/R competence skills	78 (26.2%)
Increase in knowledge about S/R	78 (26.2%)
Learning skills for addressing S/R	75 (25.2%)
Class discussions about relevant topics	62 (20.8%)
Self-reflection about R/S	34 (11.4%)
R/S completed as an aspect of multicultural competence	10 (3.4%)
*What would you like to see changed about this spiritual competency training (online and face-to-face class activities)*?	
Final case study assignment	52 (17.5%)
More religious/spiritual diversity	42 (14.1%)
More role-play activities	20 (6.7%)
More information on practical interventions	18 (6.1%)
More resources provided in the program	17 (5.7%)
Lack of time for class discussion	17 (5.7%)
*What were your primary takeaways from this spiritual competency training (online and face-to-face class activities)*?	
Importance of spiritually integrated mental health care	112 (37.6%)
Increased understanding of how to practically integrate S/R into mental health care	69 (23.2%)
Increased cultural humility in addressing S/R in mental health care	60 (20.1%)
Increased confidence in integrating S/R into mental health care	35 (11.7%)
Recognition that clinicians are allowed to discuss S/R with clients	24 (8.1%)
*Is there anything you wish you could have learned more about*?	
Information on a greater diversity of religions	67 (22.6%)
More practical strategies for implementation of S/R competence	42 (14.1%)
Ways of differentiating S/R from psychopathology	23 (7.7%)
How to utilize spiritual/religious resources in treatment	13 (4.4%)
*How do you see yourself utilizing the tools covered in this training with clients*?	
S/R assessment	134 (45.0%)
Broaching conversations about clients’ S/R	71 (23.8%)
Use of spiritual/religious resources in treatment	42 (14.1%)
S/R, religious faith and/or spirituality.	

When asked about weaknesses or possible improvements to the enhanced SCT-MH program, nearly 20% of students reported difficulties with the final case study assignment. One student stated: “*I think the case study should not be assigned at the end of the training*. *Perhaps it would be better to break the case study up into sections to be completed with the individual modules or allow more time to complete the study*.” In addition, smaller subsets of students discussed a need for greater S/R diversity, not enough practical suggestions for applying the conceptual/scientific information to clinical practice, and a desire for more downloadable resources that students could access at a later date. Regarding the first criticism, one participant shared: “*I would like to see more diversity in terms of religious and spiritual cases that gets discussed*. *At times it can seem like it is coming from a specific type of religious ideology*.”

When considering the primary takeaways from the training program, over one-third of participants discussed the general importance of attending to S/R aspects of clients’ lives. One student stated: “*Religion and spirituality play a large role in people’s lives and may play a larger role in their psychological presentation than we initially think—it is important to assess for religion/spirituality in our clients’ lives*.” In addition, roughly 20% of students noted increased understanding about how to address a client’s spirituality/religion in their mental health care and/or cultural humility and self-reflection. On the former point, one student stated: “*My primary takeaways were becoming more knowledgeable on how to use a person’s spirituality/religion as a strength and that I can support them in using spiritual practices to manage symptom[s]*.”

The last two questions focused on topics that students wished to learn more about at the end of the training and specific ways they might implement the tools with their clients. Roughly one in five students expressed a desire to learn about a more diverse group of religions; one student stated, “*I would like to have learned more in depth about different religions and cultures*. *There was a general overview*, *but I would like to learn more*. *I think maybe a follow up course would help*.” In addition, students also expressed a desire to learn more about distinguishing between spirituality/religion and psychopathology and how to implement the clinical strategies in the training program. Of the many topics and skills that are covered, nearly half of the students reported plans to incorporate S/R assessment strategies; one participant stated: “*the intake/implicit/explicit assessment questions are so helpful*. *I plan to continue utilizing those regularly now that I’ve learned them*. *Also*, *I find that being able to differentiate different spiritual/religious problems super helpful*, *and will lead to more rich work with my clients*.” In addition, 15–25% of students reported plans to simply broach or bring up the topic of spirituality/religion with their clients and help their clients to access possible strengths/resources from the clients’ spirituality/religion in the treatment process.

The attitudes, knowledge, and skills pertaining to the common intersection between spirituality/religion and clients’ mental health, lived experience, presenting problem(s), and treatment has been largely ignored in graduate training across mental health disciplines [6, 7, 25]. Without training in spiritual and religious (S/R) competencies, clinicians are more likely to neglect spiritual issues in clinical practice and fail to identify their own biases when delivering care, both of which can negatively impact the quality and utilization of mental health care [39]. To address this training need, we enhanced the SCT-MH hybrid graduate course curriculum, which can be integrated into existing clinical courses at no cost.

## Discussion

Our first hypothesis was that, when compared to changes in the six-week period without any intervention, students will report significantly greater improvements in S/R competence scores after completing the SCT-MH content. Specifically, we hypothesized that scores on self-report and objective measures of attitudes, knowledge, and skills would increase from pre- to post-training. This hypothesis was supported by the data. As expected, during the six-week control period when spirituality/religion was specifically not taught or discussed, almost no positive change was shown on any of the seven measures of S/R competence. Of note, there was slight increase on two of the skills measures and a large decrease in the third skills measures (self-efficacy) over the control period. The reason for this decline is difficult to decipher. It may be that students realized that they began the semester overconfident in their skills and that a degree of intellectual humility had settled in by mid-semester when they took Survey 2. Then, between Time 2 and Time 3 (weeks 7–12) when the SCT-MH curriculum was taught, students’ scores on all measures of S/R competence generally increased. This demonstrates that engaging with the training materials and activities appeared to promote acquisition of S/R competence in all targeted areas: attitudes, knowledge, and skills.

We included an instructor-rating of the students’ S/R competence post-intervention in the form of the graded final case study to strengthen the validity evidence supporting the intervention effect (i.e., multimethod, multi-trait measurement). We hypothesized that students’ SCT-MH final case study grade would be positively correlated with their change in S/R competence scores. We found that five of the seven S/R competence change scores were significantly positively correlated with the final case study grade: both measures of attitudes and knowledge, and one of the skill scales. Most graduate course work evaluations resemble activities like the final case study and the knowledge test; thus, it is not surprising that these measures were correlated. These findings suggest that the final case study may be a better proxy of attitudes and knowledge than of skills. A different type of assessment, such as a role play with a standardized client, may be a better assessment of students’ S/R competence skills. In addition, the five significant correlations were on the small side (.12 to .20), suggesting the concepts measured by these different assessments were related but also distinct from one another (i.e., discriminant validity evidence). Overall, the multiple measurement approaches employed in this study support inferences about the sizes and types of effects observed as a result of the SCT-MH Program.

Our second hypothesis was that the integrated curriculum approach would be a feasible, helpful, and relevant way to deliver training on S/R competence in mental health care via required coursework in graduate programs, as demonstrated by responses of students across quantitative and qualitative assessments. The results supported this hypothesis as well. Specifically, nearly all of the students planned on using spiritually integrated therapy techniques with their clients at least some of the time, found the training materials and activities helpful and relevant, and were satisfied with the online modules. Students also noted a number of strengths of the SCT-MH program, including the online materials (e.g., videos, reflection questions, case study), role plays, class discussions, framing S/R competence as a facet of multicultural competence, and increased knowledge about S/R integration in mental health care. In many cases, students who had begun the program feeling skeptical about the role of spirituality/religion in mental health ended up with a deep appreciation and understanding about the importance of addressing S/R aspects of clients’ lives in psychotherapy when appropriate. Many students noted that they felt spirituality/religion was “taboo” before this training and that they now felt they had “permission” and a greater comfort level to speak about this topic with their clients. Students reported feeling grateful for their increase in knowledge of the role that S/R plays in peoples’ lives and greater skill level in how to address a client’s S/R in their mental health care. This feedback reflects the achievement of the main goals and objectives of the SCT-MH program. An upcoming paper will report on the experience of the faculty teaching the program, which was also quite positive.

Substantive subsets of the students also identified weaknesses or possible improvements to the SCT-MH program. Namely, some students noted the length of the final case study assignment and others noted a need for greater S/R diversity, not enough practical suggestions for applying the conceptual/scientific information to clinical practice, and a desire for more downloadable resources to access at a later date. Notably, when developing the course, we made a conscious effort to include clients of diverse religious, spiritual, and non-religious/spiritual identifications in the course materials. Specifically, the case materials in the course were strategically designed to reflect the overall religious landscape of the U.S. in 2021 (i.e., the time period over which the training program was developed). At that time, 63% of Americans identified with a Christian tradition/denomination of some sort [40]. In the spirit of equitable representation, 41% (9 of 22) of the cases presented in the modules are with Christian clients from varying backgrounds. In the remainder of case examples, we presented clients from Muslim, Jewish, and Buddhist backgrounds as well as others who identify as spiritual but not religious, agnostic, and atheist. Some of the suggestions, such as more in-depth learning about diverse religions and cultures and information on how to implement the clinical strategies and approaches in the training program, might be best served by developing advanced SCT-MH training or pursuing other available resources and programs.

### Limitations

As with all research, this study was not without limitations. Of the total number of students enrolled in the 20 classes, 11% elected not to participate in the research. As such, we do not know if students who did not participate were different in any significant ways from students who did elect to participate. Like most graduate courses, we also do not know the lasting effects of the positive change in spiritual/religious competencies. Similarly, we do not know if the changes in students’ S/R competence had a favorable impact on their clinical outcomes. This is also an important direction for future research. Although we provided faculty with a detailed grading rubric and examples for calibration for the final case study, as well as training to use the rubric, we did not do a fidelity check across instructors to check accuracy and standardization of grading. Finally, the students and faculty noted a few limitations of the curriculum, including that the final case study was too long, a perception that the role plays and case study had an overly Judeo-Christian focus, and a desire for more advanced training and additional clinical topics to be included in this introductory course (e.g., sexuality, substance abuse, severe mental illness). Additional studies should also identify potential moderators, such as the religiousness of the student and mental health discipline, that may influence the effectiveness of the training.

### Strengths

This study had a number of notable strengths that bolster the conclusions we can draw from the results. These strengths include a large and diverse sample of students across mental health disciplines with a strong retention rate for this type of research across three time points; data was collected from students on their experience of the SCT-MH curriculum that allowed for a rich understanding of the use and impact of this material; use of a wait-list control group that allowed us to test for the possibility of change due to the passage of time/maturation rather than the intervention; and use of measures that assessed both subjective and objective change in S/R competence knowledge to address the possibility of subjective bias. In addition, integrating the SCT-MH content into a required semester-long clinical course gave trainees the ability to learn the material, complete clinical practice activities after each module, engage in course-based peer discussions, receive feedback from their instructors about their progress, and earn graduate-level course credits. We believe this level of integration and depth of learning will more likely lead to retention of the information and application in their clinical work. By integrating SCT-MH into required clinical courses, rather than elective courses, we also avoided the self-selection bias of only training interested students, ensuring all students received the training and developed spiritual competencies. In addition, the use of standardized curriculum and faculty training materials makes it possible for faculty with no prior experience or knowledge of the link between spirituality/religion and mental health to confidently and competently integrate SCT-MH into their courses, thus eliminating one of the major barriers to teaching S/R competencies in graduate school (i.e., lack of formal training for faculty in this area). Furthermore, by integrating the materials into part of a course, rather than attempting to add an entire course to already packed curriculums, we eliminated another barrier to this type of training in graduate school (i.e., lack of space in curriculum for new courses).

### Implications and future directions

We used the feedback from the students to make a few minor changes to the curriculum, including revising a few of the case studies so that it was clearer that they were not Jewish or Christian, adding a statement on diversity, equity, and inclusion (DEI) at the beginning of the online course, setting learning outcome expectations for a basic, rather than advanced, training in S/R competence, and reducing the number of questions in the final case study from ten to eight. All teaching materials, including access to the edX SCT-MH online course, the Instructor Guidebook, and Instructor training videos, are freely available on our website for any mental health graduate training program interested in offering the course to their students. https://www.spiritualandreligiouscompetenciesproject.com/resources/sct-mh The materials can be used flexibly and creatively, with graduate programs choosing how they want to use the SCT-MH program.

Next steps will ideally include developing training modules in advanced topics to meet the requests of many students for more training on issues such as spirituality/religion and sexuality, addictions, severe mental illness, religiously-based trauma, and specific techniques for spiritually integrated therapy. In addition, it would be beneficial to identify content in S/R and mental health to integrate into undergraduate courses, thus orienting students to this area before graduate school. Translating the SCT-MH into other languages, such as Spanish, would also increase the reach of the training materials, as well as allow us to distribute them in other countries with appropriate cultural adaptations. It will also be important to evaluate the changes in client outcomes when students and providers develop S/R competencies.

## Conclusions

Cultivating multicultural competence is a lifelong journey that requires humility and curiosity on the part of clinicians over the course of their careers. Beginning in graduate education, honoring all aspects of diversity and psychological functioning in preparation for clinical practice is crucial and demanding on faculty and trainees. Evidence from this study suggests that the enhanced SCT-MH hybrid curriculum materials can be an effective, helpful, and relevant way to address the gap in S/R competency training in graduate mental health education. By starting at the outset of graduate education, faculty and supervisors might plant the seeds of basic knowledge and expertise in the field and begin to equip a new generation of educators, clinicians, and supervisors with basic awareness, knowledge, and skills to negotiate the common intersection between S/R and mental health. Ultimately, we hope SCT-MH will be one of many contributions to a transformation in mental health practice and the psychological well-being of those we serve, as routine clinical practice becomes more adept in understanding and responding to the common S/R dimensions of human flourishing and suffering.

## Supporting information

S1 Table Covariance estimates between growth parameters. (DOCX)

S2 Table Correlation matrix for all R/S competencies. (DOCX)
