# Supplementary material for: Enhancing training in spiritual and religious competencies in mental health graduate education: Evaluation of an integrated curricular approach
Source: PLoS One. 2024 Sep 23;19(9):e0306114. doi: 10.1371/journal.pone.0306114 (PMC11419373; doi:10.1371/journal.pone.0306114)
Supplement: S1 Table — (DOCX) [file pone.0306114.s001.docx]

**Supplemental Table 1**

*Covariance Estimates Between Growth Parameters*

|  | A-S_I_ | A-S_S1_ | A-S_S2_ | SKn_I_ | SKn_S1_ | SKn_S2_ | Sk_I_ | Sk_S1_ | Sk_S2_ | SE_I_ | SE_S1_ | SE_S2_ | A-R_I_ | A-R_S1_ | A-R_S2_ | Eng_I_ | Eng_S1_ | Eng_S2_ | OKn_I_ | OKn_S1_ | Okn_S2_ |
| --- | --- | --- | --- | --- | --- | --- | --- | --- | --- | --- | --- | --- | --- | --- | --- | --- | --- | --- | --- | --- | --- |
| A-S_I_ | 3.5 |  |  |  |  |  |  |  |  |  |  |  |  |  |  |  |  |  |  |  |  |
| A-S_S1_ | -- | -- |  |  |  |  |  |  |  |  |  |  |  |  |  |  |  |  |  |  |  |
| A-S_S2_ | -0.8 | -- | -- |  |  |  |  |  |  |  |  |  |  |  |  |  |  |  |  |  |  |
| SKn_I_ | 6.1 | -- | -- | 32.1 |  |  |  |  |  |  |  |  |  |  |  |  |  |  |  |  |  |
| SKn_S1_ | -- | -- | -- | -- | -- |  |  |  |  |  |  |  |  |  |  |  |  |  |  |  |  |
| SKn_S2_ | -- | -- | 4.2 | -12.2 | -- | 22.7 |  |  |  |  |  |  |  |  |  |  |  |  |  |  |  |
| Sk_I_ | 5.3 | -- | -- | 24.5 | -- | -- | 34.8 |  |  |  |  |  |  |  |  |  |  |  |  |  |  |
| Sk_S1_ | -- | -- | -- | -- | -- | -- | -- | -- |  |  |  |  |  |  |  |  |  |  |  |  |  |
| Sk_S2_ | -- | -- | 5.0 |  | -- | 21.1 | ***2.2*** | -- | 19.1 |  |  |  |  |  |  |  |  |  |  |  |  |
| SE_I_ | 6.0 | -- | -- | 24.0 | -- | -- | 32.7 | -- | -- | 34.6 |  |  |  |  |  |  |  |  |  |  |  |
| SE_S1_ | -- | -- | -- | -- | -- | -- | -- | -- | -- | -- | -- |  |  |  |  |  |  |  |  |  |  |
| SE_S2_ | -- | -- | 4.6 | -- | -- | 24.0 | -- | -- | 27.2 | ***-2.1*** | -- | 21.8 |  |  |  |  |  |  |  |  |  |
| A-R_I_ | 4.0 | -- | -- | 10.6 | -- | -- | 14.5 | -- | -- | 14.5 | -- | -- | 25.0 |  |  |  |  |  |  |  |  |
| A-R_S1_ | -- | -- | -- | -- | -- | -- | -- | -- | -- | -- | -- | -- | -- | -- |  |  |  |  |  |  |  |
| A-R_S2_ | -- | -- | 3.4 | -- | -- | 16.0 | -- | -- | 16.2 | -- | -- | 19.4 | -8.2 | -- | 17.7 |  |  |  |  |  |  |
| Eng_I_ | 4.4 | -- | -- | 18.0 | -- | -- | 30.5 | -- | -- | 26.7 | -- | -- | 12.3 | -- | -- | 24.4 |  |  |  |  |  |
| Eng_S1_ | -- | -- | -- | -- | -- | -- | -- | -- | -- | -- | -- | -- | -- | -- | -- | -- | -- |  |  |  |  |
| Eng_S2_ | -- | -- | 3.2 | -- | -- | 12.9 | -- | -- | 19.3 | -- | -- | 18.6 | -- | -- | 12.1 | 3.2 | -- | 3.5 |  |  |  |
| OKn_I_ | ***0.0*** | -- | -- | ***0.5*** | -- | -- | ***-1.5*** | -- | -- | ***-0.2*** | -- | -- | 1.6 | -- | -- | *-1.1* | -- | -- | 1.5 |  |  |
| OKn_S1_ | -- | -- | -- | -- | -- | -- | -- | -- | -- | -- | -- | -- | -- | -- | -- | -- | -- | -- | -- | -- |  |
| Okn_S2_ | -- | -- | 0.9 | -- | -- | 5.8 | -- | -- | 4.3 | -- | -- | 6.7 | -- | -- | 5.6 | -- | -- | 3.1 | 1.2 | -- | 2.7 |

Note. “--" indicates that the covariance was not estimated and constrained to be equal to zero. Values on the diagonal are the variances of the growth parameters. Only values in italics and bold are not statistically different from zero. A-S_I_ = Attitudes (SCQ) Intercept; A-S_S1_ = Attitudes (SCQ) Slope 1; A-S_S2_ = Attitudes (SCQ) Slope 2; SKn_I_ = Subjective Knowledge Intercept; SKn_S1_ = Subjective Knowledge Slope 1; SKn_S2_ = Subjective Knowledge Slope 2; Sk_I_ = Skill (SCQ) Intercept; Sk_S1_ = Skill (SCQ) Slope 1; Sk_S2_ = Skill (SCQ) Slope 2; SE_I_ = Self-Efficacy Intercept; SE_S1_ = Self-Efficacy Slope 1; SE_S2_ = Self-Efficacy Slope 2; A-R_I_ = Attitudes (RSIPAS) Intercept; A-R_S1_ = Attitudes (RSIPAS) Slope 1; A-R_S2_ = Attitudes (RSIPAS) Slope 2; Eng_I_ = Engagement Intercept; Eng_S1_ = Engagement Slope 1; Eng_S2_ = Engagement Slope 2; OKn_I_ = Objective Knowledge Intercept; OKn_S1_ = Objective Knowledge Slope 1; OKn_S2_ = Objective Knowledge Slope 2.
