# Supplementary material for: Enhancing training in spiritual and religious competencies in mental health graduate education: Evaluation of an integrated curricular approach
Source: PLoS One. 2024 Sep 23;19(9):e0306114. doi: 10.1371/journal.pone.0306114 (PMC11419373; doi:10.1371/journal.pone.0306114)
Supplement: S2 Table — (DOCX) [file pone.0306114.s002.docx]

**Supplemental Material Table 2**

*Correlation Matrix for all R/S Competencies*

|  | 1 | 2 | 3 | 4 | 5 | 6 | 7 | 8 | 9 | 10 | 11 | 12 | 13 | 14 | 15 | 16 | 17 | 18 | 19 | 20 | 21 |
| --- | --- | --- | --- | --- | --- | --- | --- | --- | --- | --- | --- | --- | --- | --- | --- | --- | --- | --- | --- | --- | --- |
| 1 | -- |  |  |  |  |  |  |  |  |  |  |  |  |  |  |  |  |  |  |  |  |
| 2 | .43* | -- |  |  |  |  |  |  |  |  |  |  |  |  |  |  |  |  |  |  |  |
| 3 | .44* | .66* | -- |  |  |  |  |  |  |  |  |  |  |  |  |  |  |  |  |  |  |
| 4 | .39* | .67* | .72* | -- |  |  |  |  |  |  |  |  |  |  |  |  |  |  |  |  |  |
| 5 | .32* | .24* | .39* | .33* | -- |  |  |  |  |  |  |  |  |  |  |  |  |  |  |  |  |
| 6 | .32* | .55* | .70* | .73* | .39* | -- |  |  |  |  |  |  |  |  |  |  |  |  |  |  |  |
| 7 | -.06 | .07 | -.07 | -.02 | .10 | -.08 | -- |  |  |  |  |  |  |  |  |  |  |  |  |  |  |
| 8 | .53* | .39* | .34* | .28* | .36* | .29* | .09 | -- |  |  |  |  |  |  |  |  |  |  |  |  |  |
| 9 | .34* | .72* | .50* | .54* | .21* | .42* | .14* | .48* | -- |  |  |  |  |  |  |  |  |  |  |  |  |
| 10 | .25* | .47* | .64* | .54* | .35* | .56* | .04 | .39* | .65* | -- |  |  |  |  |  |  |  |  |  |  |  |
| 11 | .27* | .56* | .58* | .72* | .32* | .60* | -.03 | .39* | .65* | .72* | -- |  |  |  |  |  |  |  |  |  |  |
| 12 | .13* | .15* | .23* | .22* | .70* | .27* | .20* | .37* | .24* | .36* | .31* | -- |  |  |  |  |  |  |  |  |  |
| 13 | .22* | .37* | .55* | .50* | .38* | .60* | .02 | .32* | .49* | .75* | .67* | .40* | -- |  |  |  |  |  |  |  |  |
| 14 | -.11 | -.09 | -.15* | -.06 | .08 | -.15* | .26* | .04 | -.07 | -.13* | -.11 | .16* | -.11 | -- |  |  |  |  |  |  |  |
| 15 | .43* | .26* | .20* | .21* | .37* | .23* | -.03 | .49* | .20* | .16* | .17* | .30* | .14* | .05 | -- |  |  |  |  |  |  |
| 16 | .15* | .32* | .28* | .20* | .37* | .22* | .15* | .34* | .32* | .20* | .18* | .36* | .19* | -.04 | .47* | -- |  |  |  |  |  |
| 17 | .26* | .31* | .42* | .37* | .30* | .47* | -.07 | .30* | .29* | .44* | .34* | .27* | .41* | -.13* | .41* | .57* | -- |  |  |  |  |
| 18 | .19* | .36* | .35* | .35* | .39* | .32* | .12 | .31* | .34* | .30* | .31* | .41* | .33* | -.05 | .41* | .70* | .66* | -- |  |  |  |
| 19 | .04 | .04 | .06 | .04 | .43* | .08* | .15* | .24* | .10 | .14* | .10 | .56* | .15* | .09 | .42* | .55*** | .40* | .57* | -- |  |  |
| 20 | .16* | .31* | .41* | .38* | .31* | .47* | -.08 | .22* | .31* | .48* | .37* | .32* | .54* | -.08 | .29* | .39* | .68* | .58* | .39* | -- |  |
| 21 | -.14 | -.10 | -.24* | -.17* | .03 | -.20* | .40* | .00 | -.03 | -.21* | -.23* | .19* | -.16* | .33* | .06 | .25* | -.03 | .19* | .35* | -.04 | -- |
| 22 | -.12* | -.10 | -.09 | -.05 | .02 | -.18* | .20* | -.01 | -.01 | .00 | -.05 | .09 | -.05 | .14* | .01 | .11 | -.04 | .10 | .24* | -.10 | .35* |

1=SC Awareness: Time 1; 2=SC Knowledge: Time 1; 3=SC Skills: Time 1; 4=RSIPAS Self-Efficacy: Time 1; 5=RSIPAS Attitudes: Time 1; 6=RSIPAS Engagement: Time 1; 7=Knowledge Test Score - Time 1; 8=SC Awareness: Time 2; 9=SC Knowledge: Time 2; 10=SC Skills: Time 2; 11=RSIPAS Self-Efficacy: Time 2; 12=RSIPAS Attitudes: Time ; 13=RSIPAS Engagement: Time 2; 14=Knowledge Test Score - Time 2; 15=SC Awareness: Time 3; 16=SC Knowledge: Time 3; 17=SC Skills: Time 3; 18=RSIPAS Self-Efficacy: Time 3; 19=RSIPAS Attitudes: Time 3; 20=RSIPAS Engagement: Time 3; 21=Knowledge Test Score - Time 3; 22 = Case Study Grade

* *p* < .05
